# The role of TRPV1 channels in atherosclerosis

**DOI:** 10.1080/19336950.2020.1747803

**Published:** 2020-04-10

**Authors:** Chenyang Zhang, Lifang Ye, Qinggang Zhang, Fei Wu, Lihong Wang

**Affiliations:** a Department of Medicine, Qingdao University, Qingdao, China; b Zhejiang Provincial People’s Hospital, Qingdao University, Hangzhou, China

**Keywords:** Transient receptor potential vanilloid subfamily member 1 (TRPV1), neuropeptides, calcitonin gene-related peptide (CGRP), cardiovascular diseases, atherosclerosis

## Abstract

Transient receptor potential vanilloid subfamily member 1 (TRPV1) is a nonselective cation channel, that is mainly distributed in sensory nerve endings and can release a variety of neurotransmitters after activation. Early studies showed that it mainly conducts pain sensation, but research has demonstrated that it also plays an important role in cardiovascular diseases. Notably, in atherosclerosis, the activation of TRPV1 can regulate lipid metabolism, reduce foam cell formation, protect endothelial cells, inhibit smooth muscle cell proliferation and inhibit inflammation and oxidation. In this review, the role of the TRPV1 channel in atherosclerosis was discussed to provide new ideas for the prevention and treatment of atherosclerotic diseases.

## Introduction of TRPV1

Transient receptor potential vanilloid subfamily member 1 (TRPV1) is a cation channel belonging to the transient receptor potential (TRP) family, which has dynamic selectivity for cations such as H+, Na+, Ca2+and Mg2+. TRPV1 has a tetramer structure comprising 6 transmembrane regions, a pore-shaped region between the fifth (S5) and sixth transmembrane region (S6), a cytoplasmic amino group and a carboxyl terminus [1]. TRPV1 can be activated by nociceptive thermal stimulation (temperature> 43°C), a weakly acidic environment (pH < 6. 0), capsaicin, peptide toxin, oxygenase (ALOX), arachidonic acid metabolites, including 12 (S) hydroxyglutaric acid and 12 hydroxyhexanedienic acid (12 [S] HpETE) [2]. TRPV1 also leads to the inflow of cations, resulting in the depolarization of cells to produce an action potential, which is transmitted to the central nervous system to form a pain sensation [3]. Furthermore, the activation of TRPV1 can also promote the release of calcitonin gene-related peptide (CGRP), substance P (SP), somatostatin (SOM) and other neurotransmitters, thus playing a series of biological roles [4]. TRPV1 is mainly expressed in sensory nerve fibers, including nonmyelinated C nerve fibers and small diameter myelinated A nerve fibers [1]. Therefore, early studies focused on mainly the conduction of pain, including heat and inflammatory pain [2]. However, research has elucidated that TRPV1 is distributed in not only the nervous system, but also non-nerve tissues and organs such as the heart, liver, lung, kidney, intestine and so on [5]. In the cardiovascular system, the TRPV1 channel is distributed in the ventricle, on the epicardial surface, and in vascular endothelial cells, smooth muscle cells (SMCs) and peripheral sensory nerves of the heart [6,7]. In recent years, many studies have shown that activation of the TRPV1 channel reduces the occurrence and development of cardiovascular diseases, improves patient prognosis and plays a role in protecting the cardiovascular system.

## Introduction of atherosclerosis

**Figure 1.**
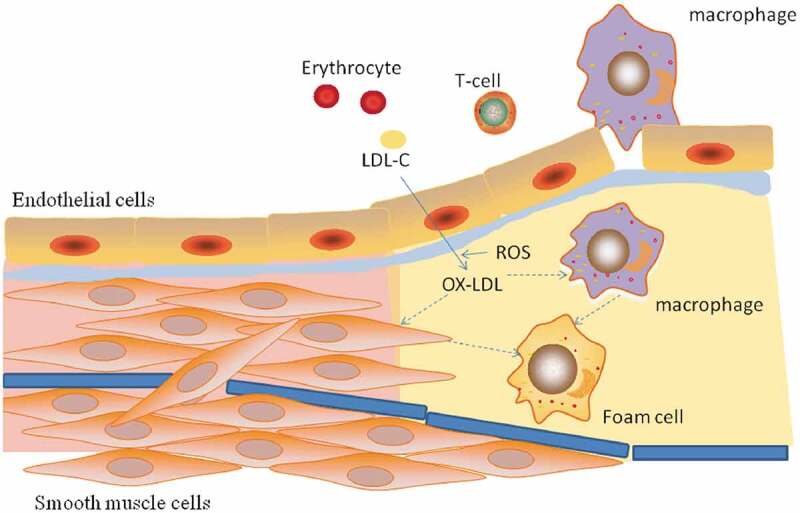
The formation of atherosclerosis. During atherosclerosis, LDL-C enters the endangium through damaged endothelial cells and is oxidized to ox-LDL-C. Macrophages enter the endangium under the action of adhesion factors and chemokines, and VSMCs proliferate and migrate from the membrane to the intima. The scavenger receptor (SR) of macrophages and VSMCs recognizes and devours ox-LDL-C to form foam cells that form early lipid streaks.

Despite tremendous advances in medicine, atherosclerosis remains the primary cause of death in both developed and developing countries. Atherosclerosis is considered to be a chronic inflammatory disease that mainly refers to large myoelastic arteries (e.g., the aorta) and medium-sized myoelastic arteries (e.g., coronary and cerebral arteries) [8].The risk factors for atherosclerosis include hypertension, hyperlipidemia, diabetes, obesity and smoking [9]. The pathogenesis mainly includes endothelial cell injury, lipid deposition, foam cell formation, inflammation, oxidative stress, and SMCs proliferation, among others [10] (Figure 1).

## Relationship between TRPV1 and atherosclerosis

### Regulation of lipid metabolism

The most important factor underlying atherosclerosis is abnormal lipid metabolism, especially hypercholesterolemia, for which low-density lipoprotein cholesterol (LDL-C) is an acknowledged risk factor. LDL-C enters the vascular intima through damaged endothelial cells and is converted to oxidized low-density lipoprotein cholesterol (ox LDL-C), which can be recognized by the scavenger receptor (SR) of vascular smooth muscle cells (VSMCs) and monocyte-derived macrophages and then engulfed to form foam cells and early lipid streaks [10] (Figure 1).

TRPV1 can regulate lipid metabolism, initially by reducing blood lipid levels, and Ma et al., who fed capsaicin to atherosclerotic mice, showed that compared with those in ApoE^(−/−)^TRPV1^(−/−)^mice, the serum total cholesterol and triglyceride levels in apolipoprotein E knockout mice (ApoE^(−/−)^) mice were significantly reduced, and lipid accumulation in aortic SMCs was also significantly reduced [11]. In terms of its mechanism, Li et al. showed that TRPV1 is expressed in liver cells. After activation of the TRPV1 channel, the expression of uncoupling protein 2 (UPC2) in the liver is increased, the β-oxidation of fat is accelerated, and the serum triglyceride and liver fat levels are decreased in wild-type (WT) mice, while TRPV1 knockout mice exhibit no significant changes [12]. Ohyama K et al. showed that capsaicin can increase the oxidative decomposition of fat and inhibit obesity by increasing sympathetic nerve activity and catechin fenamine release [13]. Furthermore, after activating the TRPV1 receptors, capsaicin can reduce the inflammation of adipose tissue and increase the secretion of adiponectin by adipocytes, which can increase insulin sensitivity and further regulate the metabolism of glucose and fat [14].

In addition, TRPV1 is expressed in autonomic nerve center, which can regulate glucose and lipid metabolism and energy balance by regulating autonomic nervous system. First of all, TRPV1 receptors are expressed in the hypothalamus paraventricular nucleus (PVN) which can adjust the feeding behavior, digestive function and glucose homeostasis [15,16]. Studies have shown that a high-fat diet can reduce the expression of TRPV1 receptors, while capsaicin increases the expression of TRPV1 and anorexic genes and reduces the expression of orexigenic genes in the hypothalamus of mice on a high-fat diet [17].The hypothalamus TRPV1 activation can adjust the diet behavior through the gastric-related PVN neurons releasing glutamic acid, and regulate the liver metabolism through the liver-related PVN neurons [18,19]. Second, TRPV1 is also expressed in brainstem vagus nerve dorsal complex, and it can regulate the vagal output to the subdiaphragmatic organs to regulate gastric function, liver metabolism and insulin secretion [16,20].

**Figure 2.**
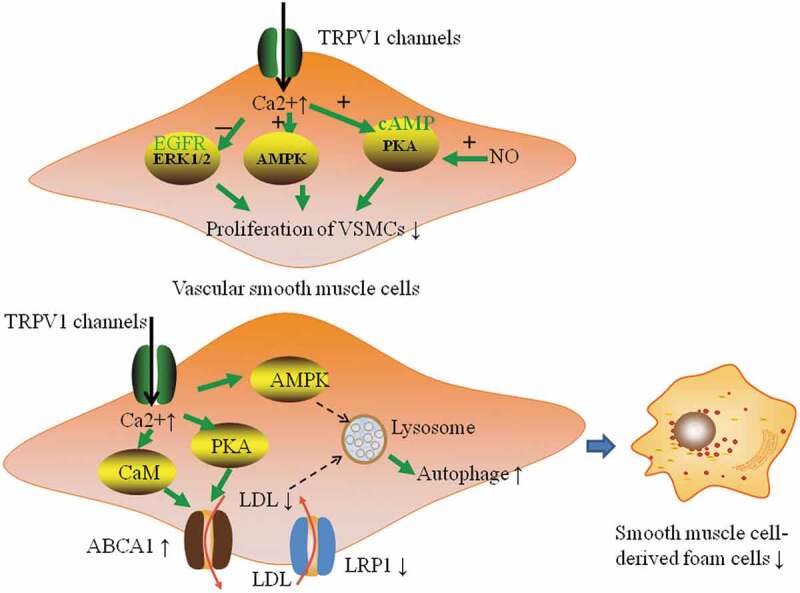
Effect of TRPV1 on the proliferation of VSMCs and the formation of smooth muscle cell-derived foam cells. Activation of TRPV1 channels in smooth muscle cells can increase Ca^2+^ inflow, increase the expression of ATP binding box transporter A1 (ABCA1, cholesterol efflux regulatory protein) and reduce the expression of low-density lipoprotein-related protein 1 (LRP 1, cholesterol inflow regulator protein) through CaM- and PKA-dependent mechanisms to promote the outflow of LDL and reduce VSMC lipid storage. It can also activate the autophagy-lysosomal pathway of VSMCs through the AMPK signaling pathway to recover autophagy damage by ox-LDL and ultimately inhibit the formation of foam cells. In addition, activation of TRPV1 can inhibit the proliferation of VSMCs through the cAMP/PKA pathway, and NO can enhance this effect by increasing the cAMP level. TRPV1 activation can inhibit the proliferation and migration of VSMCs by activating the antigrowth adenosine 5ʹ-monophosphate-activated protein kinase (AMPK) signaling pathway. It can also inhibit the Ang II-induced proliferation and migration of VSMCs by deactivating the epidermal growth factor receptor (EGFR)-ERK 1/2 pathway.

TRPV1 can also reduce aortic lipid accumulation and reduce foam cell formation. Foam cells derived from SMCs are the most abundant cells in human atherosclerotic lesions, accounting for at least 50% of the atherosclerotic lesion cells [21,22]. VSMCs express many cholesterol uptake receptors and reverse cholesterol transporters, including LDL receptor- related protein 1 (LRP 1), ATP binding box transporter A1 (ABCA 1) and low-density lipoprotein (LDL) receptor [23,24]. Among them, ABCA 1 can promote the outflow of intracellular cholesterol, while LRP1 can promote the uptake of cholesterol by cells. Ma et al. showed that capsaicin-activated TRPV1 could increase ABCA 1 expression in aortic VSMCs and reduce the LRP 1 expression and lipid storage in the VSMCs of ApoE^(-/-)^ mice through calcium-dependent, calmodulin- and protein kinase A(PKA)-dependent mechanisms, but these effects were not observed in ApoE^(-/-)^TRPV1^(-/-)^ mice [11] (Figure 2). In addition, studies have shown that the formation of VSMC-derived foam cells is associated with SIRT1 damage and enhanced nuclear factor-kB (NF-kB) activity. SIRT1 is an antiarteriosclerotic factor that can regulate cholesterol metabolism and reduce the inflammatory response [25]. TRPV1 activation by capsaicin can inhibit the formation and migration of VSMC-derived foam cells by protecting SIRT 1 and inhibiting NF-kB signaling [26]. In macrophage-derived foam cells, Zhao et al. showed that TRPV1 agonists could upregulate the expression of ABCA1 and ABCG1 in macrophages by regulating liver X receptor α-(LXRα-)-dependent transcription, which could promote the outflow of cholesterol from macrophages, thus delaying the development of atherosclerosis [27].

### Induction of autophagy

Autophagy mainly involves the formation of double-membrane autophagosomes, which are fused with lysosomes and degraded [28]. Previous studies have shown that TRPV1 is closely related to autophagy. After activation, TRPV1 can induce the autophagy of thymus cells through the adenosine 5ʹ-monophosphate-activated protein kinase (AMPK) and Atg4C pathways regulated by reactive oxygen species (ROS). Among them, Atg4C is a cysteine protease that is believed to mediate the execution of autophagy [29]. In cultured hypoxic cardiomyocytes, TRPV1 can increase the expression of autophagy-associated proteins such as lysosomal associated membrane protein 1 (LAMP-1) and LAMP-2 through the autophagy-lysosomal pathway, thus increasing the autophagy of the cells and thereby reducing the damage of early hypoxic cardiomyocytes [30].

Autophagy plays a vital role in promoting the outflow of cholesterol, reducing the formation of foam cells, and inhibiting inflammation, and can thus reduce atherosclerosis to a certain extent [31]. Ouimet, M. et al. suggested that macrophages engulf lipid droplets, which are then delivered to lysosomes and subsequently hydrolyzed by lysosomal acidic lipase, and cholesterol is then expelled from macrophage foam cells through an ABCA 1-dependent pathway [32]. Activation of TRPV1 by capsaicin can activate the autophagy – lysosomal pathway of VSMCs through the AMPK signaling pathway to restore autophagic factors damaged by ox-LDL and ultimately inhibit the formation of foam cells [33] (Figure 2). Studies have shown that as photothermal switches for TRPV1 signaling, copper sulfide (CuS) nanoparticles can increase the number of opened TRPV1 channels and the intracellular Ca^2+^ content, activate autophagy in oxLDL-treated VSMCs and prevent the formation of foam cells [34].

### Improvement of vascular endothelial injury

Vascular endothelial injury is the initiating factor of atherosclerosis. LDL-C enters the intima of the vascular wall through the damaged endothelium and is oxidized to ox-LDL-C, aggravating endothelial injury. Studies have shown that the mRNA expression of TRPV1 in injured vascular endothelial cells is significantly lower than that in intact endothelial cells, suggesting that TRPV1 is related to vascular endothelial function [35].

**Figure 3.**
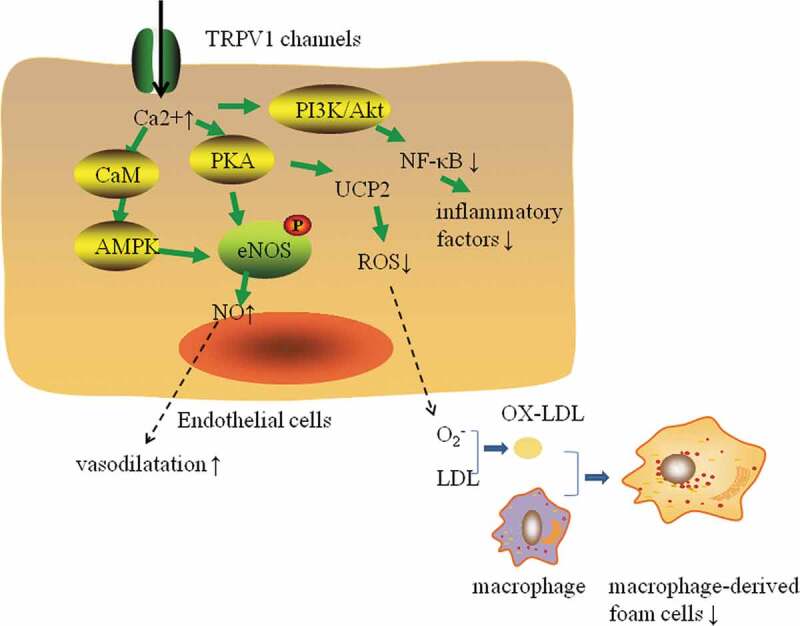
Effect of TRPV1 channels on the protection of ECs and the formation of macrophage-derived foam cells. Activation of TRPV1 channels in endothelial cells can increase intracellular Ca^2+^ levels and promote PKA, CaMKII and AMPK activation. PKA and AMPK can promote eNOS phosphorylation and increase the level of NO, which can promote vasodilation and angiogenesis. Activation of PKA can upregulate uncoupling protein 2 (UCP2) expression, reduce ROS production, increase resistance to oxidative stress, and reduce endotheliocyte damage, foam cell formation and atherosclerosis. In addition, activation of TRPV1 channels can activate the Ca^2+^-dependent phosphatidylinositol 3 kinase (PI3K)/Akt pathway to inhibit the activation of NF-B, which can inhibit cytokines, chemokines, adhesion factors and monocyte adhesion to inhibit the inflammatory response of ECs.

Nitric oxide (NO) is an oxidant produced by endothelial cells and macrophages that plays an important protective role in atherosclerosis, but this role mainly involves the NO produced by endothelial nitric oxide synthase (eNOS) [8]. In contrast, NO produced by high-volume inducible nitric oxide synthase (iNOS) in macrophages has a strong antioxidant function and no protective effect on atherosclerosis. Knowles et al. found that eNOS^(−/−)^ApoE^(−/−)^ mice could exhibit hypertension and atherosclerosis, while iNOS^(−/−)^ApoE^(−/−)^ mice rarely developed atherosclerosis [36]. Yang et al. found that the TRPV1 channel activation induced by capsaicin could upregulate the expression of PKA in endothelial cells and promote the phosphorylation of eNOS, thus improving vascular endothelial function and increasing the release of NO [37]. Ching’s study showed that after excitation of TRPV1 by evodiamine, the AMPK and eNOS phosphorylation in aortic endothelial cells increased, which promoted the formation of the TRPV1- eNOS complex, increased the production of NO, promoted the formation of new blood vessels, and delayed the generation of atherosclerosis in mice [38]. In addition, the study found that TRPV1 activation in endothelial cells could increase the Ca2+concentration and the expression of calmodulin kinase -II (CaMKII). The combination of Ca^2+^ and CaMKII can promote the activation of eNOS, induce NO production and promote vasodilatation and angiogenesis [39] (Figure 3).

ROS can also cause endothelial cell damage, further aggravate the development of atherosclerosis. It has been found that short-term hydrogen peroxide stimulation could activate TRPV1 channels to increase coronary artery vasodilatory blood flow in mice. As the stimulation time increased, the activity of TRPV1 gradually decreased, and intracascular ROS and lipid levels would increase [40]. ROS can activate inflammatory factors to induce monocytes and lymphocytes to adhere to endothelial cells and to enter the intima of vessels, promoting the occurrence and development of atherosclerosis [41]. In atherosclerosis mice, capsaicin actives PKA, increases UCP2 expression, reduces ROS production, resists oxidative stress, protects endothelial cells and improves endothelial function damage [42,43] (Figure 3).

### Inhibition of inflammation

Atherosclerosis is a chronic inflammatory process and inflammatory cytokines are involved in the whole process of atherosclerosis formation.

Previous studies have shown that activation of TRPV1 can inhibit inflammation and that TRPV1 can inhibit the activation of NF-κB and inflammation through a variety of mechanisms. Wei et al. found that the levels of serum cholesterol, tumor necrosis factor a (TNF-a), interleukin 6 (IL-6), monocyte chemoattractant protein 1 (MCP-1) and macrophage inflammatory protein 2 (MIP-2) were higher in ApoE^(−/−)^TRPV1^(−/−)^ mice than in ApoE^(−/−)^ mice [44]. Mechanisms underlying these increased expression levels may include the following: activation of TRPV1 downregulates epidermal growth factor receptor (EGFR) levels by inducing its ubiquitination and degradation, thereby inhibiting the EGFR/mitogen-activated protein kinases (MAPKs) pathway. MAPKs are a group of serine/threonine protein kinases composed of multiple isoenzymes (ERK, P38, and JNK) [45]. Inhibition of any of the three enzymes reduces the expression levels of cytokines and adhesion molecules [46], especially those involved in the ERK pathway, which is closely related to various inflammatory responses [47]. In addition, activation of TRPV1 can activate the Ca^2+^-dependent phosphatidylinositol 3 kinase (PI3K)/Akt pathway and the downstream eNOS/NO pathways, inhibit the generation of cytokines, chemokines and adhesions and mononuclear cell adhesion by inhibiting the activation of NF-κB to suppress the inflammatory response of vascular endothelial cells (ECs) [48] (Figure 3).

In addition, CGRP released after the activation of TRPV can also inhibit inflammation and regulate the balance of inflammation to a certain extent via the following possible mechanisms: first, CGRP receptors are composed of mainly a calcitonin receptor-like receptor (CLR) and a receptor activity modifying protein (RAMP), and RAMP 1 is a specific subunit of the CGRP receptor. RAMP 1-deficient mice were shown to exhibit higher blood pressure levels than WT mice. CGRP was shown to effectively dilate the arteries of WT mice and decrease the production of TNF-α and IL-12 in dendritic cells (DCs) stimulated by lipopolysaccharide (LPS), but this effect was not observed in RAMP 1 deficient mice, indicating that CGRP can regulate vasodilation and reduce inflammatory mediators through CLR/RAMP 1 [49]. Second, CGRP can activate eNOS and cyclooxygenase-1 (COX-1) in vascular endothelial cells to promote the production of NO and prostaglandin, both of which can inhibit TNF transcription by inhibiting the activation of NF-kB and thus play an anti-inflammatory role [50,51]. Third, Zhang et al. inserted the autologous rabbit jugular vein grafts transfected with CGRP gene into the carotid artery to simulate arterial reconstruction, and found that CGRP could inhibit macrophages from releasing inflammatory mediators such as MCP-1, TNF-a, iNOS, matrix metalloproteinase-9 (MMP-9) and reduce VSMC proliferation and intimal hyperplasia [52]. Forth, in vascular endothelial cells, CGRP can significantly reduce the production of chemokines CXCL 1, CXCL 8 and CCL 2 by inhibiting the activation of NF-kB, thus reducing the aggregation of monocytes and neutrophils to the vascular wall [53]. It can also regulate the immune function of monocytes/macrophages, inhibit the degranulation of mastocytes and inhibit the antigen presentation of DCs [54].

### Inhibition of smooth muscle cell proliferation

During the formation of atherosclerosis, VSMCs migrate from the membrane to the intima, proliferate significantly under the action of thrombin, and synthesize proteoglycan, collagen and elastin to form a plaque matrix [8].

Activation of TRPV1 can reduce VSMC proliferation and vascular remodeling. CGRP released by peripheral nerves can inhibit the growth of aortic VSMCs in rabbits and rats by increasing cyclic adenosine phosphate (cAMP), thereby counteracting the stimulation of IL-1 and TNF to the proliferation of VSMCs [55]. Similarly, Via in vitro experiments, Chattergoon indicated that CGRP can inhibit the proliferation of cultured aortic and VSMCs through the cAMP/PKA pathway, and nitric oxide (NO) has been shown to enhance the antiproliferative effect of CGRP in VSMCs by increasing the content of cAMP [56,57]. In addition, studies have shown that TRPV1 inhibits the proliferation and migration of VSMCs, and vascular remodeling by activating the antigrowth AMPK signaling pathway [58]. CGRP can inhibit Ang II-induced rat aortic SMC proliferation by deactivating the EGFR-ERK 1/2 pathway [59,60]. Recent studies have also shown that CGRP-modified mesenchymal stem cells inhibit the phenotypic regulation and proliferation of VSMCs [61]. Endogenous CGRP inhibits oxidative stress and the proliferation and migration of VSMCs in the presence of vascular injury [60].

### Inhibition of apoptosis

The apoptosis of VSMCs accelerates the development of atherosclerosis, and eNOS deficiency leads to the increased susceptibility of VSMCs to proapoptotic damage. Especially compared with those in WT mice, the expression levels of aggrecan in the aortic tissues of eNOS-deficient mice were significantly increased. Aggrecan is a proteoglycan which is selectively deposited in the intima of advanced atherosclerotic lesions and induces VSMC apoptosis. This result suggests that eNOS deficiency may increase aggrecan expression and promote the apoptosis of VSMCs, thus promoting the progression of atherosclerosis [62].Activation of TRPV1 channels can promote the phosphorylation of eNOS and increase the release of NO [37]. CGRP may also inhibit the activation of CaMKII and the downstream transcription factor CREB and inhibit Ang II-induced ROS-dependent VSMC apoptosis [63]. Studies have also shown that CGRP can antagonize ROS-induced VSMC apoptosis through the ERK1/2 and MAPK pathways [64].

### Lowering of blood pressure levels

Hypertension is an important risk factor for atherosclerosis and can aggravate vascular endothelial cell injury. Studies have shown that activation of TRPV1 can reduce blood pressure, and the antihypertensive mechanism of TRPV1 may include the following: (1) after activation of TRPV1 in peripheral nerves, neuropeptides such as CGRP and SP are released to relax blood vessels and produce antihypertensive effects [65]. Among these peptides, CGRP can dilate blood vessels and reduce blood pressure by increasing intracellular NO and K^+^ concentrations via endothelium- and nonendothelium-dependent mechanisms [37,66]. (2) Activation of TRPV1 can reduce the renal perfusion pressure, increase the glomerular filtration rate, and reduce the expression of epithelial sodium channels (α-ENaC), thereby inhibiting Na^+^ and water reabsorption and reducing blood volume and blood pressure [67]. (3) TRPV1 itself can act as an intravascular baroreceptor to changes in mechanical pressure and thus play a role in blood pressure regulation [68]. (4) Activation of TRPV1 can reduce ROS, resist oxidative stress, protect ECs, improve endothelial function and regulate blood pressure [41]. (5) CGRP may reduce blood pressure by antagonizing the renin-angiotensin-aldosterone system (RAAS) and the sympathetic nervous system [69,70]. (6) CGRP can promote the separation of endothelin-1 (ET-1) from the ETA receptor, thereby terminating the vasoconstriction of ET-1, lowering blood pressure and inhibiting the hypertrophy and proliferation of VSMCs [71].

### Regulation of glucose metabolism

Diabetes is an equal-risk coronary heart disease, and many patients with diabetes suffer from diffuse coronary stenosis [72]. Elevated blood sugar levels can cause endothelial damage, chronic inflammation and nerve damage, and abnormal blood glucose metabolism is often accompanied by obesity and high blood lipid levels, which are risk factors for atherosclerosis.

TRPV1 activation can reduce blood glucose levels and improve the vascular damage caused by high glucose levels. Studies have shown that the TRPV1 agonist capsaicin can increase the production of CGRP, the expression of glucose transporter 4 (GUT4) and glucose uptake by increasing the expression of SIRT6, thereby lowering blood glucose levels [73]. When mice with type 2 diabetes mellitus (T2DM) were administered an analog of CGRP, their circulating glucagon-like peptide-1 (GLP-1) level increased, their fasting blood glucose, fasting insulin and glycosylated hemoglobin (HbA1c) levels decreased, and their food intake and body weight decreased significantly [74]. After activation, TRPV1 can control islet inflammation and regulate insulin resistance and leptin resistance [75,76]. And it can also regulate oral glucose tolerance and correct the dysfunction of insulin secretion by reducing low-grade inflammation of T2DM [77]. In addition, through the ERK/HIF-1/VEGF signaling pathway, CGRP can inhibit hyperglycemia-induced angiogenesis, reduce intracellular ROS levels, change eNOS mRNA expression, and protect mouse vascular ECs from hyperglycemic damage [78]. CGRP also plays a protective role in the oxidative damage of rat aortic endothelial cells (RAECs) induced by high glucose levels by inhibiting ERK 1/2-NOX4 [79]. Studies have also shown that SP rather than CGRP can promote the proliferation of human pancreatic duct cells through the NK1R and Wnt signaling pathways, but these duct cells do not differentiate into β-cells. A lack of SP may be the cause of diabetes, and SP can also be used to treat diabetes [80].

## Conclusion

In summary, TRPV1 activation can improve atherosclerosis through a variety of mechanisms, but its clinical application is focused on mainly analgesia. Although capsaicin can activate TRPV1 channels and reduce lipid storage and atherosclerosis formation, its chronic toxicity limits its clinical application. However, it has been indicated that the coupling of CuS nanoparticles with a TRPV1 antibody is similar to the photothermal switch of infrared light in VSMC TRPV1 channels. After irradiation, the thermal sensitivity of the TRPV1 channels increased, causing a large influx of Ca^2+^, activation of ox-LDL-induced VSMC autophagy and prevention of foam cell formation. In vivo, CuS-TRPV1 allows for the photoacoustic imaging of cardiac vessels and reduces lipid storage and plaque formation [34]. Although some progress has been made, the clinical application of TRPV1 in the protection of atherosclerosis needs to be further explored.
